# Realization of p-type gallium nitride by magnesium ion implantation for vertical power devices

**DOI:** 10.1038/s41598-019-45177-0

**Published:** 2019-06-19

**Authors:** Ya-Ting Shi, Fang-Fang Ren, Wei-Zong Xu, Xuanhu Chen, Jiandong Ye, Li Li, Dong Zhou, Rong Zhang, Youdou Zheng, Hark Hoe Tan, Chennupati Jagadish, Hai Lu

**Affiliations:** 1 School of Electronic Science and Engineering, Nanjing University, Nanjing, 210093 China; 2 Department of Electronic Materials Engineering, Research School of Physics and Engineering, The Australian National University, Canberra, ACT 2601 Australia; 3 Collaborative Innovation Center of Advanced Microstructures, Nanjing University, Nanjing, 210093 China; 4 Australian National Fabrication Facility, Research School of Physics and Engineering, The Australian National University, Canberra, ACT 2601 Australia

**Keywords:** Materials for devices, Electrical and electronic engineering

## Abstract

Implementing selective-area p-type doping through ion implantation is the most attractive choice for the fabrication of GaN-based bipolar power and related devices. However, the low activation efficiency of magnesium (Mg) ions and the inevitable surface decomposition during high-temperature activation annealing process still limit the use of this technology for GaN-based devices. In this work, we demonstrate successful p-type doping of GaN using protective coatings during a Mg ion implantation and thermal activation process. The p-type conduction of GaN is evidenced by the positive Seebeck coefficient obtained during thermopower characterization. On this basis, a GaN p-i-n diode is fabricated, exhibiting distinct rectifying characteristics with a turn-on voltage of 3 V with an acceptable reverse breakdown voltage of 300 V. Electron beam induced current (EBIC) and electroluminescent (EL) results further confirm the formation of p-type region due to Mg ion implantation and subsequent thermal activation. This repeatable and uniform manufacturing process can be implemented in mass production of GaN devices for versatile power and optoelectronic applications.

## Introduction

GaN is an excellent candidate for high-performance power devices owing to its superior properties such as large bandgap, high electrical breakdown field, high electron mobility and relatively high thermal conductivity^1–5^. Until recently, lateral device architecture based on AlGaN/GaN heterojunctions is the mainstream of power device development, which has been commercialized by several companies with good success. Compared to lateral devices, vertical GaN power devices have several potential advantages such as higher current density, smaller footprint and lower dynamic on-resistance^6–8^. However, to realize high performance vertical devices, effective termination structures, such as guard rings or junction termination extension^9,10^, are necessary, which would require selective area p-type doping technique. Regrowth of GaN has been attempted for selective area p-type doping but regrowth processes are complex, which result in interfacial problems and low breakdown in power devices^11,12^.

Commonly used in the mature fabrication technology of Si and SiC power devices, ion implantation provides an attractive alternative for selective doping in the fabrication of vertical GaN power devices. It can also simplify the fabrication process of termination structures with precise doping control and highly compatible with complementary metal oxide semiconductor (CMOS) technology. However, ion implantation in group III-nitride materials, especially for p-type doping, has been shown to be significantly challenging^13–15^. Sufficiently high annealing temperature is required for recovery of crystalline damage induced by ion bombardment and efficient activation of Mg acceptors. Annealing at temperatures higher than 900 °C could also result in serious decomposition of GaN. To overcome this effect, multicycle rapid thermal annealing (MRTA) process has been demonstrated as an attractive method to enable a high Mg activation ratio while minimizing decomposition of GaN^16,17^. However, MRTA technique is fairly complicated and not commercially available. It has also been reported that the use of a single (e.g. AlN, Si_3_N_4_, SiO_2_) or multiple (e.g. AlN/SiO_2_, AlN/Si_3_N_4_, AlN/SiN_x_) capping layers^16,18,19^ can prevent damage and decomposition of the implanted GaN during high-temperature annealing. However, subsequent removal of Si_3_N_4_ or AlN capping layer after thermal annealing is an issue^14^ due to crystallization of the layer. Furthermore, although SiO_2_ or Si_3_N_4_ protective layer is a good choice for Si implantation to enhance n-type conductivity of GaN, they are not suitable for p-type doping. Therefore, a thorough consideration on choosing the proper protective layer is essential not only for suppressing implantation damage but also for easy removal after the high temperature activation process. These unresolved challenges therefore, hamper the development of GaN-based vertical power devices. In addition, although there have been several reports of Mg ion implantation of GaN, conclusive evidence showing the formation of p-type GaN is still lacking.

In this work, we develop an effective and simple thermal annealing method to realize p-type GaN by employing proper protective layers during ion implantation and post implantation annealing. An AlN capping layer grown by atomic layer deposition prevents amorphization and roughing of the topmost GaN surface due to implantation-induced damage caused by Mg ions; while a SiO_2_ protective layer suppresses decomposition of GaN at high annealing temperatures. By using these optimal processes, the formation of p-type GaN by Mg ion implantation could be achieved, as evidenced by the positive Seebeck coefficient extracted from thermopower measurements. To further verify the p-type conductivity, GaN p-i-n diodes were fabricated by Mg implantation. These devices show excellent rectifying characteristics with a forward turn-on voltage of 3 V (consistent with the bandgap of GaN) and a reverse breakdown voltage of over 300 V. The spatial distribution of electron beam induced current (EBIC) across the junction and the observation of ultraviolet emission by means of electroluminescence (EL) at forward bias also validate the realization of p-type GaN by ion implantation.

## Ion Implantation and Thermal Annealing

The epitaxial layer structure used in this work consists of 1 μm unintentionally doped (UID)-GaN, 2 μm Si doped n-GaN with an electron concentration of ~5 × 10^18^ cm^−3^, and 4 μm UID-GaN, grown on sapphire substrate by metal-organic chemical vapor deposition (MOCVD). The experimental details of GaN growth can be found in the Supplementary Information (Section 1). Mg ion implantation was performed at an incident angle of 7° off the surface normal at room temperature (RT) or high temperature (HT, 300 °C) with two energy steps: 30 keV with a dose of 1.5 × 10^14^ cm^−2^ and 60 keV with a dose of 2 × 10^14^ cm^−2^, giving rise to an average Mg concentration of ~5 × 10^18^ cm^−3^ within a projected range of 300 nm according to secondary ion mass spectroscopy (SIMS) measurements. Post-implantation annealing was performed in nitrogen ambient for 30 minutes at 1100, 1230, 1300 or 1400 °C. As shown in Supplementary Fig. S1, the implanted samples annealed at 1300 and 1400 °C suffer serious decomposition even when covered by a 200-nm-thick SiO_2_ protective layer. Although the sample after annealing at 1100 °C is somewhat transparent, Seebeck results still present a negative coefficient, indicating that the implanted Mg ions have not been successfully activated at this temperature. In comparison, at an annealing temperature of 1230 °C, the implanted layer becomes transparent and exhibits a much smoother surface, suggesting that the implantation-induced damage has largely been annealed with minimal surface decomposition, consistent with previous reports^14,18^. Consequently, annealing at 1230 °C for 30 minutes in N_2_ was then used for annealing throughout this work.

To evaluate the influence of the protective capping layers used for ion implantation or annealing, identical epitaxial layer structures were prepared and treated in different ways as listed in Table 1, and the detailed flow chart for the process can be found in Supplementary Fig. S2. The as-grown GaN epilayer without ion implantation was annealed at 1230 °C for 30 mins in N_2_, which is the reference sample denoted as Sample A. Samples B, C, D and F were implanted at RT, while Sample E was implanted at 300 °C. Prior to implantation, a 20-nm thick AlN layer was deposited on Samples D, E and F by plasma enhanced atomic layer deposition (PEALD) at 250 °C to reduce the implantation damage. After that, the AlN layer was removed by developer solution (ZJX-100). Prior to the annealing, a 200-nm thick SiO_2_ encapsulation layer was deposited on Samples C, D and E by plasma enhanced chemical vapor deposition (PECVD) at 350 °C to suppress surface decomposition during the high-temperature annealing. After annealing, the SiO_2_ capping layer was removed using buffered oxide etch (BOE) for the subsequent fabrication and characterization of the p-i-n diodes.

**Table 1:** Summary of implantation temperatures and protective layers used for Samples A–F.

Sample ID	Implantation Temperature	AlN	SiO_2_	Sample ID	Implantation Temperature	AlN	SiO_2_
A	—	—	—	D	RT	√	√
B	RT	—	—	E	HT	√	√
C	RT	—	√	F	RT	√	—

### SiO_2_ protection layer for high-temperature annealing

**Figure 1:**
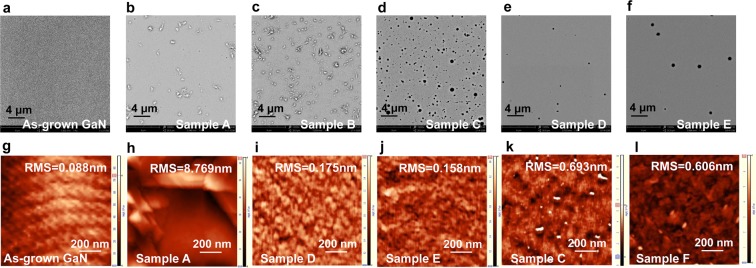
Top-view SEM (**a**–**f**) and AFM (**g**–**l**) images of the as-grown GaN sample and Samples A–F investigated in this study.

Figure 1a–f are scanning electron microscopy (SEM) micrographs of the samples to evaluate the influence of the protective capping layers during implantation and annealing. All images are taken on GaN surface after removal of the capping layers. For Samples A and B, the scattered “white spots” are seen on the surface, indicating that the annealing process at high temperature in the absence of SiO_2_ protection layer result in obvious decomposition of the GaN surface layer. The rougher surface in Sample B is due to the implantation-induced damage. Energy-dispersive spectrometry (EDS) (see Section 5 in the Supplementary Information) results from the “white spots” of Sample B show the compositional ratios of O and N are 32% and 23%, respectively, implying the presence of residual oxygen in the annealing chamber. After HCl (HCl:H_2_O = 1:1) treatment at a temperature of 60 °C for 30 mins, the white spots can be partially removed accompanied by the appearance of dark pits and the O composition decreased to 1.5%; whereas the reserved white bulges on the surface of Sample B could be due to recrystallization of GaN during the high temperature annealing. In comparison, SEM image of Sample C (Fig. 1d) protected by 200 nm SiO_2_ layer during annealing exhibits a relatively smooth surface after the removal of SiO_2_ cap, except for the presence of dark spots. EDS measurements inside and outside the dark spots show the composition as Ga:N≈ 41%:59% and 37%:63%, respectively, while Si and O are not detectable. It suggests that the SiO_2_ encapsulation layer can effectively suppress nitrogen escaping during the high temperature annealing process, and the diffusion of Si or O from SiO_2_ capping layer into GaN is negligible due to the strong Si-O bonds and low diffusion coefficient of oxygen^20^. Besides the SiO_2_ capping layer, Si_3_N_4_ and AlN have been reported as a protective layer for the annealing process after Mg ion implantation^16,18^. However, the crystallization of Si_3_N_4_ and AlN cause difficulties in the subsequent removal by simple wet etching after thermal annealing^14^. We have also attempted to use Si_3_N_4_ as the capping layer during annealing and as expected, it can only be removed by plasma etching, resulting in a rough surface which is not beneficial for the formation of a good Ohmic contact.

### AlN protection layer for Mg ion implantation

To reduce the surface damage by ion implantation, a 20-nm thick AlN layer was deposited on Sample D before implantation, in comparison to Sample C which did not have the AlN layer. From Fig. 1d,e, one can clearly see that the AlN layer can provide a remarkable improvement in surface morphology. The density of the dark pits estimated from SEM images is reduced by more than one order of magnitude from 5.3 × 10^7^ cm^−2^ for Sample C to approximately 3.6 × 10^6^ cm^−2^ for Sample D. It was explained that the presence of a thin AlN capping layer prevents the formation of a nanocrystalline surface layer during the implantation^21^, as the AlN capping layer causes more nuclear collisions and absorbs the implantation damage^22^. Especially for terminal devices, another unique advantage of AlN capping layer with a proper thickness is to enable most implanted-ions concentrating right at the surface of the target GaN sample, which is beneficial to realize high hole concentration at the topmost region and fascinate the formation of Ohmic contact with low specific-on resistance.

### High-temperature Mg ion implantation

Dynamic annealing, a self-recovery process, simultaneously accompanies the damage creation process caused by implantation and this process is further enhanced at higher implantation temperature. Indeed, high temperature implantation is reported to be favorable for lattice recovery and improve the activation of Mg acceptors in GaN^23^. We compare Mg implantation at 300 °C (Sample E) with implantation carried out at room temperature (Sample D). Comparing the surface morphologies of these two samples the dark pits in Sample E decrease in density but increase in size, as shown in Fig. 1e,f. This suggests that the implantation-induced defects may have merged and evolved into extended defects during implantation at higher temperature and reduced in density, as a result of stronger dynamic annealing.

Atomic force microscope (AFM) images of Samples C–F are shown in Fig. 1i–l to quantify the surface roughness of the various samples. For the reference Sample A (as-grown GaN) before annealing, an atomic step-flow feature with a root-mean-square (RMS) roughness of 0.088 nm was measured (Fig. 1g), indicating the high quality of the as-grown GaN epilayer. Figure 1i,j are the corresponding AFM images of Samples D and E after implantation, annealing and removal of the protective layers, with a surface roughness of 0.175 and 0.158 nm, respectively. The atomic step-flow feature appears to have been destroyed by ion implantation and annealing, and low density of tiny voids are observed, which increases in size for Sample E. As shown in Fig. 1k,l, the surface of Samples C (w/o AlN protective layer for implantation) and F (w/o SiO_2_ protective layer for annealing) are relatively rough with an RMS value of 0.693 and 0.606 nm, respectively. These results emphasize the significant role of the protective capping layers during implantation and annealing.

### Evolution of chemical states and Fermi level shift

**Figure 2:**
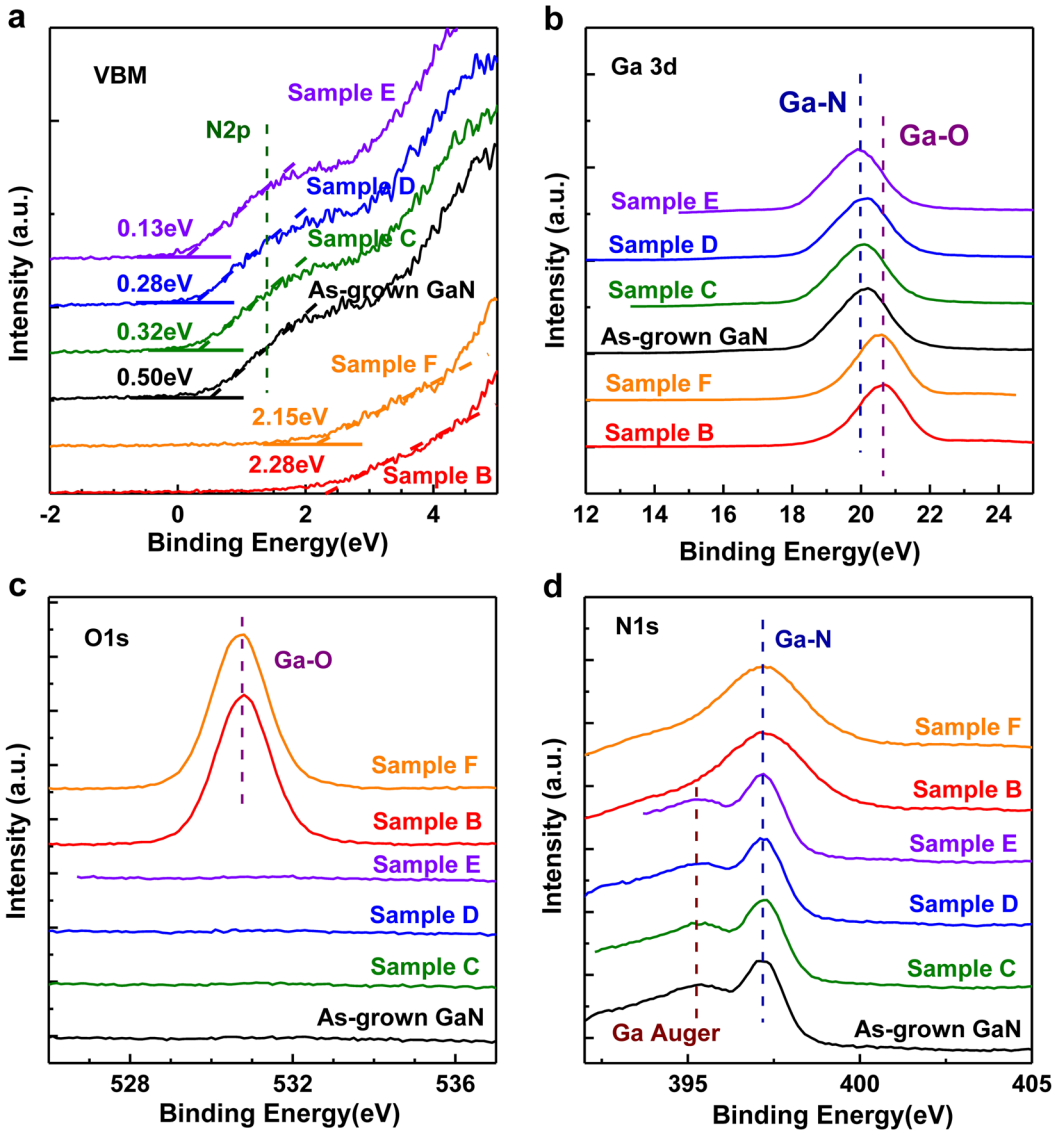
Valence band edge spectra (**a**), Ga3d peaks (**b**), O1s peaks (**c**), and N1 s peaks (**d**) of the as-grown GaN sample and Samples B–F.

To confirm the effective role of the protective layers on the creation of p-type GaN, X-ray photoelectron spectroscopy (XPS) measurements were employed to characterize all samples in this work. Before XPS, all the samples were immersed in BOE for 5 mins to ensure that the surface native oxide has been removed. The XPS spectra of the valence band and Ga 3d, O 1 s and N 1 s core levels are shown in Fig. 2a–d, respectively. All XPS spectra have been calibrated against the C1s peak at 284.6 eV as a reference. As shown in Fig. 2a, the shoulder corresponding to the N2p electronic states can be clearly identified in the as-grown GaN sample and Samples C–E with SiO_2_ capping layer during high temperature annealing, while it is almost gone in Samples B and F which have been annealed without any capping layer. Meanwhile, Ga3d and O1s core-level spectra in Fig. 2b,c confirm the existence of Ga-O bonding in Samples B and F. In Fig. 2d, Ga-N bonding as well as Ga Auger peaks can be observed in the as-grown GaN sample and Samples C–E, while Samples B and F only have Ga-N bonds. These results indicate that the inevitable decomposition of GaN and oxidization caused by residual oxygen in the furnace in samples without SiO_2_ protection. In comparison, the SiO_2_ encapsulation layer on Samples C–E can effectively prevent nitrogen escaping and oxygen in-diffusion during the high-temperature annealing process, which is correspond to the SEM and EDS results.

The variation of conduction can also be evaluated from the Fermi level shift with respect to the valence band maximum (VBM). Figure 2a shows the normalized VB XPS spectra of the undoped GaN and Samples B–F. The zero point corresponds to the location of the Fermi level. In the presence of N2p electronic states, the uppermost valence band edge (VBE) can be resolved, and the VBM is located at the intersection point between the linear fitting line and the background. It is found that the value of E_F_-E_V_ decreases from 0.50 eV for the as-grown GaN sample to 0.13 eV for the implanted Sample E, implanted and annealed with protective of capping layers. The Fermi level approaches VBM in Samples C–E indicating effective p-type doping through optimized implantation and thermal activation processes with proper capping layers.

### Secondary ion mass spectroscopy (SIMS) and X-ray diffraction (XRD) (Depth profiling and structural properties)

**Figure 3:**
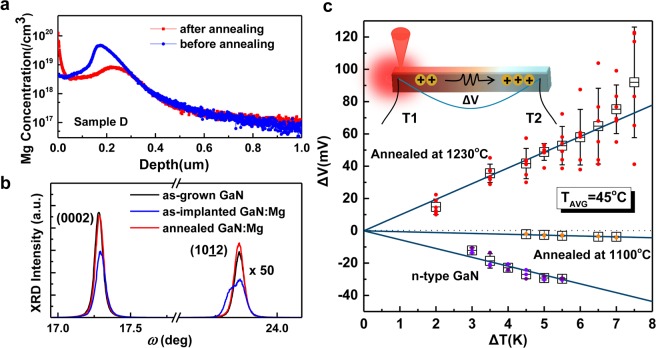
(**a**) SIMS data of Mg implanted GaN sample (Sample D) before and after annealing. (**b**) XRD rocking curves of ω-scan corresponding to the (0002) and (10–12) planes of as-grown GaN and Sample D before (as-implanted GaN:Mg) and after annealing (annealed GaN:Mg). (**c**) Thermopower results of GaN:Mg annealed at 1230 °C and 1100 °C. The results from an n-type GaN are also shown for comparison. The inset shows a schematic of thermopower measurement setup.

Figure 3a shows the SIMS data of the implanted Sample D before and after annealing. The blue curve is the result from Sample D right after ion implantation (AlN removed), and the red one refers to the sample after annealing (SiO_2_ removed). Due to the diffusion of Mg in GaN, there is a redistribution of the Mg profile after annealing, with a strong accumulation towards the surface, which forms a heavily doped region that is beneficial for Ohmic contact formation. Figure 3b displays the XRD rocking curves of ω-scan corresponding to the GaN (0002) and (10–12) planes of Sample D before and after annealing, as well as the as-grown GaN epilayer for reference. The reduced intensity and broad XRD peaks in the as-implanted GaN layer is a result of lattice disorder induced by implantation and the asymmetric feature with a slight shift is due to strain caused by the presence of Mg and defects within the implanted layer. The broadened feature of the asymmetric (10–12) plane indicates the introduction of in-plane lattice distortion due to implantation^24^. Upon annealing with the SiO_2_ capping layer, the shape and width of the XRD patterns are almost same as those of the as-grown GaN epilayer, indicating removal of the accumulated strain and implantation-induced damage. More XRD results can be found in Section 4 in the Supplementary Information.

### Seebeck coefficient

Hall effect measurement is the most popular method to evaluate carrier transport properties including the carrier type, concentration and mobility. However, the measurement brings difficulty to precisely determine the transport properties in the multi-layers, in which two kinds of carriers may participate in the transport and more than one conductive channels occur. Especially in this work, as discussed in Supplementary Information (Section 7) due to the large difference in carrier concentration, mobility and thickness of multilayers, p-type conduction, which is formed in the shallow surface region with a rather small distributed thickness and low mobility, might be compensated or masked by the stronger n-type conduction of the underlying unimplanted n-type GaN region. Temperature-dependent Hall effect results on the implanted and undoped GaN samples are shown in Supplementary Fig. S6. The carrier concentration of Mg-implanted GaN film after thermal annealing is lower than the undoped GaN film and the difference between electron concentration becomes larger with the elevated temperature and a decrease of carrier concentration is observed at a “kink” temperature point around 540 K. It is an indirect evidence of the creation of mobile holes as a result of Mg activation in the implanted region.

Alternatively, thermopower measurement is an effective method to determine the conduction type of semiconductors as reported in^25–28^. It has been applied to demonstrate p-type carriers where Hall effect measurement fails as in the case of InN which shows parallel n-type carrier conduction^26,27^. Thermopower measurements were performed, as illustrated by the schematic in the inset of Fig. 3c. K-type thermocouples were used to determine the temperature gradient between two Ohmic contacts on the samples. The Seebeck voltage was measured between two thermocouples. To avoid contribution of n-type conduction from the 2 μm-thick Si-doped layer, an alternative set of samples with only 1.5 μm UID-GaN were grown on sapphire by MOCVD for this parallel study. The samples were implanted with Mg ions at either RT or 400 °C with two energies: 250 keV at a dose of 4 × 10^14^ cm^−2^ and 500 keV at a dose of 6 × 10^14^ cm^−2^. The samples were then annealed at 1100 or 1230 °C for 30 minutes in N_2_. During implantation and annealing, identical AlN and SiO_2_ protective layers as Sample D were used as described above. Mg concentration distribution above 5 × 10^18^ cm^−3^ to a depth of ~0.63 μm is obtained from SIMS measurement as shown in Supplementary Fig. S5. This profile is slightly shallower than that calculated by SRIM, which may be due to the out-diffusion of Mg towards the surface region during annealing. The thermopower measurement results are shown in Fig. 3c. For the sample implanted at 400 °C and annealed at 1230 °C, a positive Seebeck coefficient is obtained at an average measurement temperature of 45 ± 5 °C. It provides direct evidence of the existence of free holes and conversion to p-type conduction^25,26^. For sample implanted at RT and annealed at 1230 °C similar results are also obtain (see Supplementary Fig. S11). For comparison, the measured Seebeck coefficient of an as-grown n-type GaN is also plotted. As expected, the sign is negative. For the samples that were implanted at RT (yellow dots in Fig. 3c) or 400 °C (see Supplementary Fig. S11) and annealed at 1100 °C, both show a reduced negative Seebeck coefficient. It can be concluded that there is insufficient activation of Mg acceptors upon annealing at 1100 °C, resulting in an incomplete conversion to p-type material.

## P-i-n Diode Characterization

**Figure 4:**
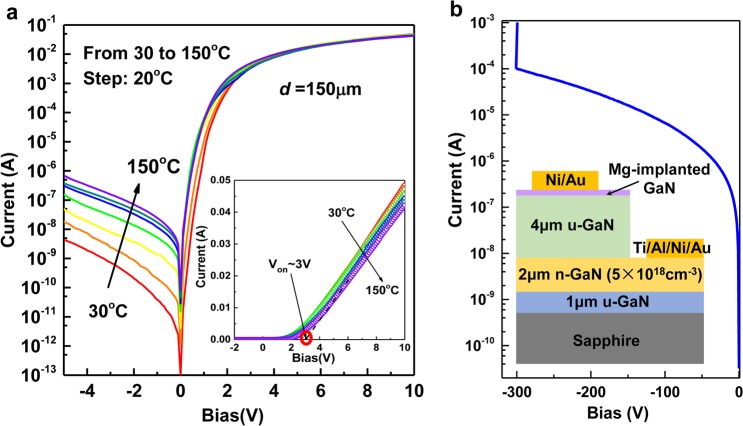
Electrical characterization of the Mg implanted GaN p-i-n diode. (**a**) Temperature-dependent current-voltage (I-V) characteristics. The inset shows the data plotted on a linear scale to determine the turn-on voltage (3 V). (**b**) Reverse breakdown characteristics. The inset is the cross-sectional schematic of the fabricated GaN p-i-n diode.

On the basis of the above process optimization, p-i-n diodes composed of the p-type GaN implanted layer, the remaining UID GaN layer and the bottom n-type GaN, were fabricated based on Sample D to verify the implementation of vertical GaN-based power devices. The cross-sectional schematic of the GaN p-i-n diode is shown in the inset of Fig. 4b. Details of the fabrication process can be found in Section 8 in the Supplementary Information.

### Current transport characterization

Figure 4a shows the temperature-dependent current-voltage (I-V) characteristics of the vertical GaN p-i-n diode. The device exhibits excellent rectification behaviour with an on-off current ratio of ~10^7^ at ±4 V at room temperature. With increasing temperature from 30 to 150 °C, the reverse current leakage increases, which can be attributed to enhanced tunneling assisted processes caused by traps or defects along the vertical direction^29^. In the forward current region (>4 V), the turn-on current decreases with increasing temperature due to increased resistance in the drift region caused by reduced electron mobilities resulting from increased phonon scattering. As shown in Supplementary Fig. S7, Ohmic contacts are formed at both n- and p-type sides of the device, and thus, the rectifying feature is an indisputable indication of the formation of a p-n junction through Mg ion implantation^9,14,18,30^. From the linear plot in the inset of Fig. 4a, the turn-on voltage is around 3 V, consistent with the bandgap of GaN. The other important feature in Fig. 4b is a breakdown voltage of 300 V with a peak electric field of 2.8 MV/cm at the edge of p-i-n junction as simulated using Silvaco (see Supplementary Fig. S9). Although it is below the theoretical limit of GaN (about 3.8 MV/cm), the performance is still acceptable. To further improve the performance of the device, the formation of dark pits after implantation and annealing needs to be addressed and eliminated.

### Electron beam induced current (EBIC) characterization

**Figure 5:**
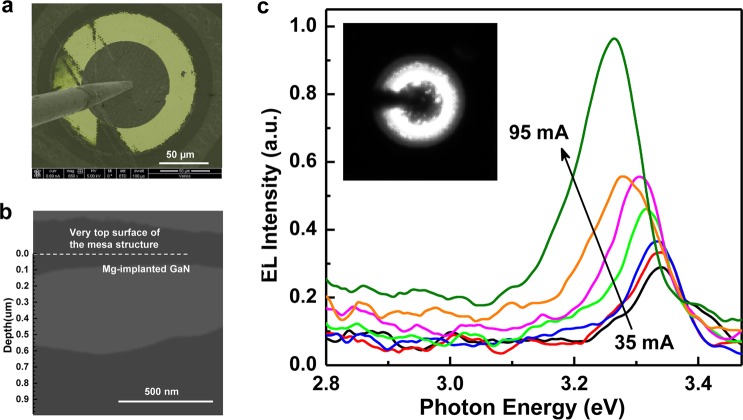
(**a**) Top-view composite SEM-EBIC map of the Mg ion-implanted p-i-n diode taken at an acceleration voltage of 10 kV and a beam current of 0.69 nA at RT and zero bias. (**b**) 60° tilted cross-sectional view of the EBIC map at RT and zero bias. (**c**) Electroluminescence (EL) spectra of the Mg implanted GaN p-i-n diode at RT under different injection currents. The insets show the EL image at 95 mA injection current.

To provide further evidence of p-type formation by Mg ion implantation, EBIC measurements were performed at zero bias at room temperature. The EBIC signal was recorded simultaneously with the SEM detector signal, thereby allowing us to spatially correlate the EBIC signal. The electron beam direction was perpendicular to the surface of the p-i-n junction and the acceleration voltage was varied from 1 to 15 kV. Figure 5a shows the top-view composite SEM-EBIC image at an acceleration voltage of 10 kV and beam current of 0.69 nA. This corresponds to a penetration depth of ~400 nm in the sample^31^. The yellow color region is the current generated inside the depletion region where electron-hole pairs induced by the electron beam are separated by the internal electric field and collected by the contacts. It therefore provides further evidence of the formation of a p-n junction^32,33^. Figure 5b shows the 60° tilted cross-sectional view of the EBIC map of the junction. A higher EBIC signal detected in the range of 100 to 600 nm beneath the surface, which correspond to the i-layer, where the built-in field is the driving force for the collection of excess carriers generated by the electron beam.

### Electroluminescence (EL) characterization

The EL spectra of the Mg ion-implanted sample were measured at room temperature to identify the presence of p-i-n junction in our device (see Fig. 5c). At a forward injection current of 35 mA, a peak emission at the wavelength of 372 nm (3.34 eV) could be observed at the edge of the circular electrodes as shown in the inset of Fig. 5c. Due to the thick UID layer sandwiched in the p-i-n diode, excess electrons and holes injected from the n- and p-GaN layer, respectively, into the i-layer lead to a strong band-to-band radiative recombination. When the bias current is increased from 35 to 95 mA, the emission intensity is enhanced together with a slight red-shift in the emission peak from 3.34 eV (372 nm) to 3.26 eV (381 nm). The temperature-dependent bandgap shrinkage is a feature due to thermal effects at high injection current level as previously reported^34^. The light output-current (L-I) characteristics can be fitted by the power law, *P* = *cI*^*m*^, where parameter *m* reflects the effect of deep level states on the recombination process. The fitting in Supplementary Fig. S10 gives rise to a value of *m* = 2, indicating non-radiative recombination via residual defects that are not fully annealed is still dominant. Nevertheless, the presence of EL UV emission together with typical L-I characteristics of an LED provides a direct evidence of the formation of p-i-n structure.

## Conclusion

In summary, we demonstrate p-type doping on GaN through Mg ion implantation and a subsequent thermal activation processes with proper protective layers. By choosing AlN and SiO_2_ as the capping layers during ion implantation and high-temperature annealing, respectively, the reduction of lattice damage, the effective suppression of decomposition/oxidization and inter-diffusion as well as the easy removal of the capping layers can be achieved. These combined processes result in smooth surface morphology and easy formation of an Ohmic contact to the implanted region. P-i-n diodes for vertical power devices are also demonstrated with an excellent rectifying characteristic, a turn-on voltage of 3.0 V and a breakdown voltage of 300 V. The carrier transport and recombination processes in the p-i-n diode are also revealed by spatially correlated EBIC measurements and electroluminescence. Our processes using a combination of Mg ion implantation and protective capping technology provide an easy strategy to realize full potential of GaN in power electronic devices with CMOS compatible implantation processes.

## Supplementary information

Supplementary Information
