# Supplementary material for: Realization of p-type gallium nitride by magnesium ion implantation for vertical power devices
Source: Sci Rep. 2019 Jun 19;9:8796. doi: 10.1038/s41598-019-45177-0 (PMC6584666; doi:10.1038/s41598-019-45177-0)
Supplement: Supplementary file 1 — Supplementary Information [file 41598_2019_45177_MOESM1_ESM.pdf]

Supplementary Information

Realization of p-type gallium nitride by magnesium ion implantation for vertical power devices

Ya-Ting Shi[1,3], Fang-Fang Ren[1,3], Wei-Zong Xu[1], Xuanhu Chen[1,3], Jiandong Ye[1], Li Li[4], Dong Zhou[1], Rong Zhang[1,3], Youdou Zheng[1,3], Hark Hoe Tan[2], Chennupati Jagadish[2] and Hai Lu[1,3]

[1] *School of Electronic Science and Engineering, Nanjing University, Nanjing 210093, China*

[2] *Department of Electronic Materials Engineering, Research School of Physics and Engineering, The Australian National University, Canberra, ACT 2601, Australia*

[3] *Collaborative Innovation Center of Advanced Microstructures, Nanjing University, Nanjing 210093, China*

[4] *Australian National Fabrication Facility, Research School of Physics and Engineering, The Australian National University, Canberra, ACT 2601, Australia*

Correspondence and requests for materials should be addressed to F.-F. R. (ffren@nju.edu.cn) or H. L. (hailu@nju.edu.cn)

1. Experimental details of GaN growth

The epitaxy of GaN and *in-situ* Si n-type doping were performed by metal-organic chemical vapor deposition (MOCVD) technique with the following procedures. GaN was grown at 1050 ℃ on (0001) sapphire substrates with a trimethylgallium (TMGa) flow of 96 μmol/min and an ammonia flow of 89 mmol/min at 200 mbar in hydrogen atmosphere with a total gas flow of 7.5 slm. Silane (SiH_4) was used as in-situ doping sources. All samples were grown and consist of AlN/AlGaN seed and buffer layers followed by a thick undoped GaN buffer layer. For the thin GaN epilayer, the thickness of undoped GaN is 1.5-μm, while for the p-i-n diode fabrication, the implantation were performed on a 7-μm-thick GaN epilayer, which consists of 1-μm unintentional doped (UID)-GaN, 2-μm Si doped n-GaN (electron concentration of ~5×10^{18} cm^{-3}), and 4-μm UID-GaN.

2. Ion implantation and optimum thermal activation

For the 7-μm-thick GaN epilayer, a 20-nm-thick AlN capping layer was deposited by plasma enhanced atomic layer deposition (PEALD) at 250 ℃ on top of GaN. Subsequently, Mg ion implantation process was carried out at room temperature (RT) or high temperature with an incidence angle 7 °off the normal surface in two steps: an accelarating energy of 30 keV with a dose of 1.5×10^{14} cm^{-2} and an energy of 60 keV with a dose of 2×10^{14} cm^{-2}. After implantation, the AlN capping layer was removed by immerging it in developer solution (ZJX-100) for 30 mins.

For Seebeck coefficient measurements, the alternative samples with a thinner UID-GaN (1.5μm) grown on sapphire by MOCVD was chosen for parallel implantation study to avoid the contribution of n-type conduction from the 7μm-thick GaN samples. The alternative samples were implanted in two steps with the individual condition of 250 keV with a dose of 4×10^{14} cm^{-2} and 500 keV with a dose of 6×10^{14} cm^{-2}. The higher implantation energy and dose make Mg concentration to be ~10^{18} cm^{-3} in the deep depth of ~0.9μm estimated by SRIM.

To optimize the thermal activation process for both the recovery of crystalline damages induced by implantation and the electrical activation of Mg acceptors, we performed annealing in nitrogen ambient for 30 minutes at different temperatures, including 1100, 1230, 1300 and 1400 ℃. Prior to the thermal activation process, a 200-nm-thick SiO_2 encapsulation layer was deposited on the implanted sample by plasma enhanced chemical vapor deposition (PECVD) at 350 ℃ to suppress the surface decomposition and oxidation during the high-temperature annealing. SiO_2 capping layer was removed after annealing by using buffered oxide etch (BOE, HF 14% concentration). The morphological evolution of implanted layers were evaluated after the removal of SiO_2 layer and shown in Figure S1(a-f). The insets in panels (c-e) are the corresponding SEM images to clarify the details of surface damages.

To further demonstrate the effect of thermal process on the activation of optical properties of Mg-implanted GaN, optical transmission spectra of samples are measured by a UV-visible near-IR scanning spectrophotometer (Lambda 950, PerkinElmer) at room temperature and plotted in Figure S1(g) for comparison. As expected, the transmission of the as-implanted GaN is reduced due to the presence of sub-gap states induced by abundant defects after implantation. After anealing at 1100 or 1230 ℃ , the transmittance properties are effectively recovered as compared to that of the as-grown GaN, indicating that optical activation process is almost completed. However, for the sample annealed at 1100 ℃, the Seebeck results still present negative coefficient, which is shown in Figure 3 in the main manuscript, indicating that electrical activation process is not fully completed due to not high enough temperature.

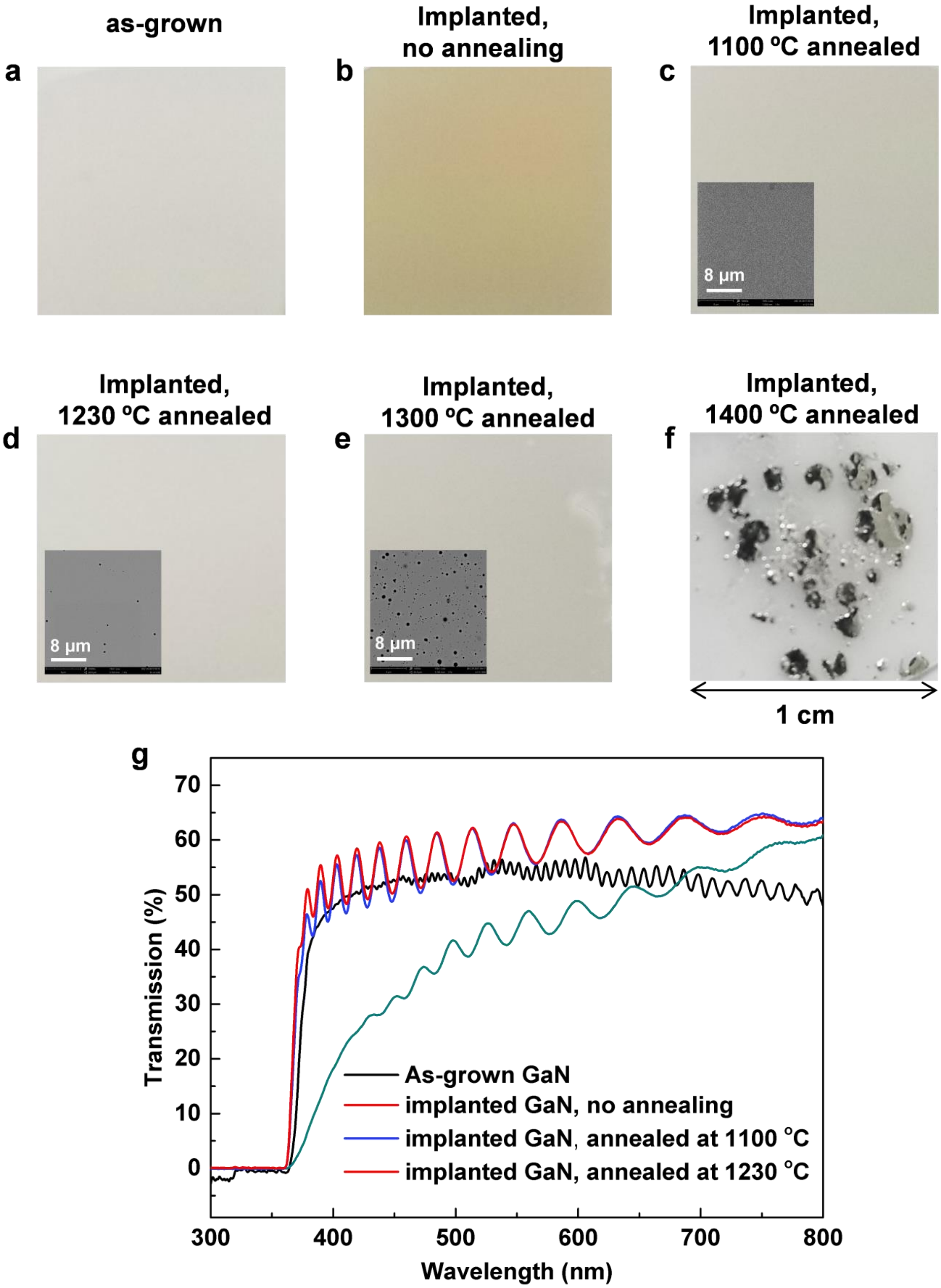

Figure S1. Top-view photographs of (a) as-grown GaN epilayer, (b) Mg-implanted GaN before post-annealing, (c-f) Mg-implanted GaN annealed at 1100, 1230, 1300 or 1400 °C, respectively. (g) Measured optical transmission spectra of the as-grown GaN epilayer, Mg-implanted GaN before post-annealing, Mg-implanted GaN annealed at 1100 or 1230 °C, corresponding to the samples shown in panels (a-d) respectively.

3. Detailed flow chart of the as-grown GaN and Samples A-F

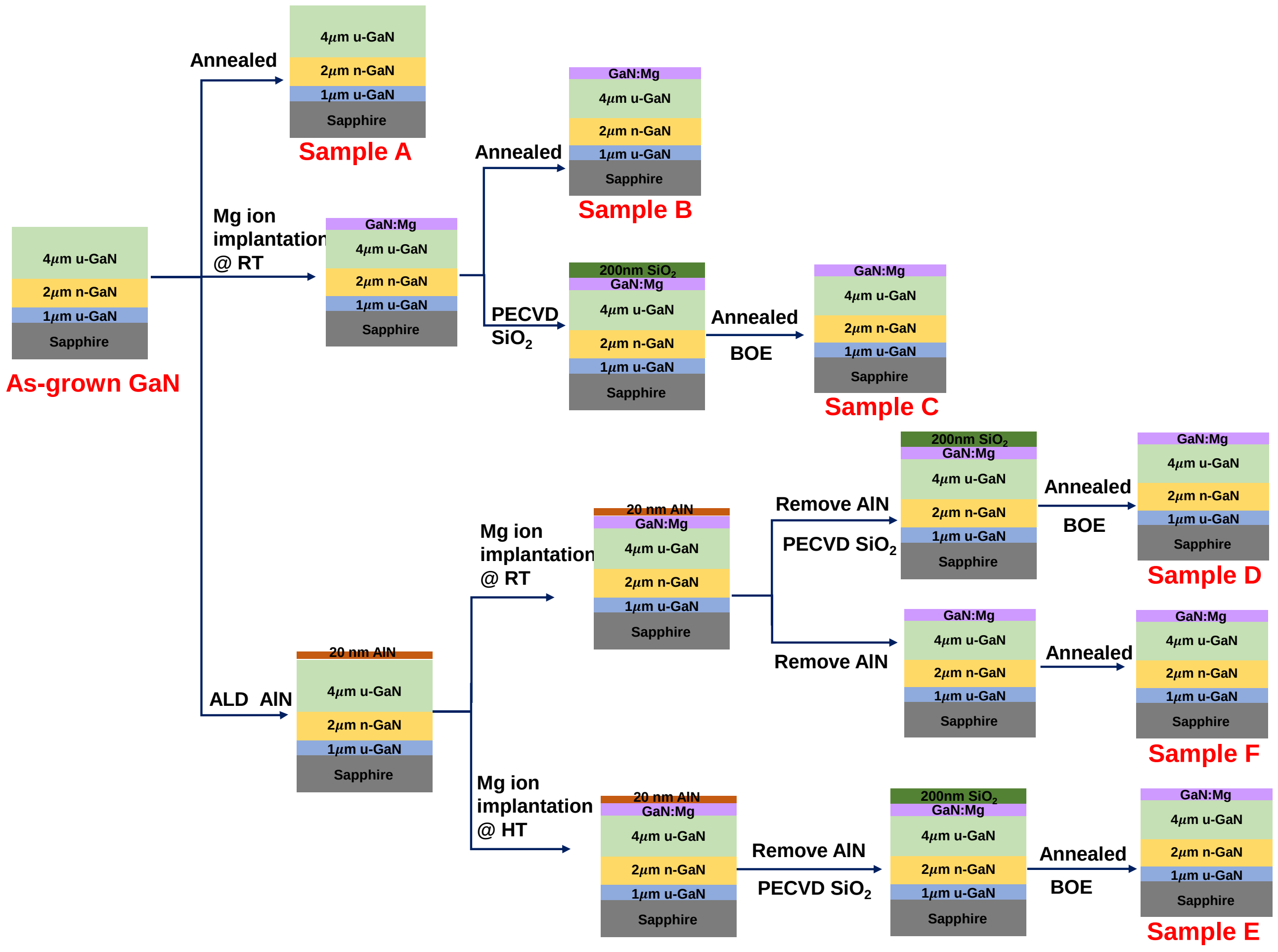

Figure S2. Detailed flow chart of the as-grown GaN and Samples A-F under various manufacturing processes.

A detailed flow chart of samples A-F under various manufacturing processes mentioned in the manuscript can be found in Figure S2. As shown in the flow chart, a 20-nm-thick amorphous AlN capping layer was only deposited on Samples D, E and F (not on other samples) by plasma enhanced atomic layer deposition (PEALD) at 250 ℃ with 300 cycles. After implantation, the amorphous AlN capping layer was removed by the developer solution (ZJX-100) for 30 mins. To suppress the surface decomposition during the high-temperature annealing, a 200-nm SiO_2 encapsulation layer was deposited on Samples C, D and E by plasma enhanced chemical vapor deposition (PECVD) at 350 ℃ prior to the annealing. After annealing process, the SiO_2 capping layer was removed by using buffered oxide etch (BOE), and then the samples were used for the subsequent electrical and thermopower characterization

and as well the fabrication of p-i-n diodes. Samples A-F were all annealed at 1230 ℃ for 30 mins in N_2. Besides, Samples B, C, D and F were implanted at RT, while Sample E was implanted at 300 ℃ (HT). Sample B-F have the same implantation energy and dose (30 keV with a dose of 1.5×10^{14} cm^{-2} and 60 keV with a dose of 2×10^{14} cm^{-2}).

4. High-resolution X-ray diffraction (HRXRD)

Table S1. HRXRD results of samples at different conditions.

					(0002)		(10-12)	
					Peak(θ)	FWHM(“)	Peak(θ)	FWHM(“)
As-grwon GaN					17.2837	234.72	24.0438	295.56
400 ℃ implanted	RT implanted	Annealed Temp(℃)	AlN	SiO_2				
√			√		17.2923	246.96	24.0366	446.76
	√		√		17.2922	260.64	24.0129	485.64
	√	1100	√		17.2857	238.32	24.0459	283.32
	√	1100	√	√	17.2854	241.6953	24.0447	290.5943
√		1230			17.2842	228.24	24.0404	287.64
√		1230	√		17.2844	227.88	24.0397	293.76
	√	1230	√		17.2828	225	24.0433	290.88
	√	1230			17.2826	238.32	24.0442	288.72
√		1230	√	√	17.2855	244.44	24.0436	330.5783
	√	1230	√	√	17.2851	237.6804	24.0448	290.9064
	√	1300	√	√	17.2868	243	24.0475	317.16
√		1300	√	√	17.2847	242.8989	24.0451	310.2782

The microstructures of implanted samples are evaluated by high resolution X-ray diffraction (HRXRD) using a D8 advance system with a Cu Kα X-ray source and a high resolution of 0.0001°. As summarized in Table S1, high dose ion implantations do cause inevitable damages to the materials. The observed broadening and shift of diffraction peaks indicates the prescence of strain and lattice disorder. After high temperature annealing, the full-width at half maximum (FWHM) of diffraction peaks are reduced to be almost same as the as-grown sample, indicating that the themal activation process can effectively heal the lattice damages and relax the strain. In addition, the results also confirm the significance of the AlN and SiO_2 protective layers during the implantation and thermal activation

processes, respectively. Details of the difference between variable samples in SEM and AFM morphologies are also verified through HRXRD results.

5. Energy dispersive spectrometer (EDS) characterization

In this work, a detailed investigation was carried on the protection role of SiO_2 layer during the high-temperature annealing. Based on the energy dispersive spectrometer (EDS) measurements and the X-ray photoelectron spectroscopy (XPS) measurements, we found that the SiO_2 encapsulation layer can effectively suppress the formation of oxide/oxygen-enriched materials and also prevent the nitrogen escaping during the high-temperature annealing process. The suppressed diffusion of donor-like impurities in GaN is critical for the conversion from n- to p- type conduction. Figure S3 shows the SEM images of Sample B and Sample C. The difference between these two samples is that a 200-nm SiO_2 is coated on Sample C as the protecting layer during thermal annealing while Sample B has no protection. The details can be seen in Figure S2. The EDS results of the red spot on Sample B exhibit ~32% oxygen and ~23% Nitrogen. After HCl ($HCl:H_2O=1:1$) treatment at a temperature of 60 ℃ for 30 mins, the white spots on Sample B can be partially removed accompanied by the appearance of dark pits and the O composition decreased, as shown in Figure S4. It indicates that without any protection, the decomposition of GaN and surface oxidization is serious, which is also confirmed by the XPS measurement. While for Sample C, after the removal of SiO_2, the EDS results exhibit ~60% Nitrogen and 40% Gallium, while Si and O are not detectable, confirming the effective protection by SiO_2 layer during high temperature annealing. These results are consistent with the results of XPS measurement as shown in Figure 2 in the main manuscript.

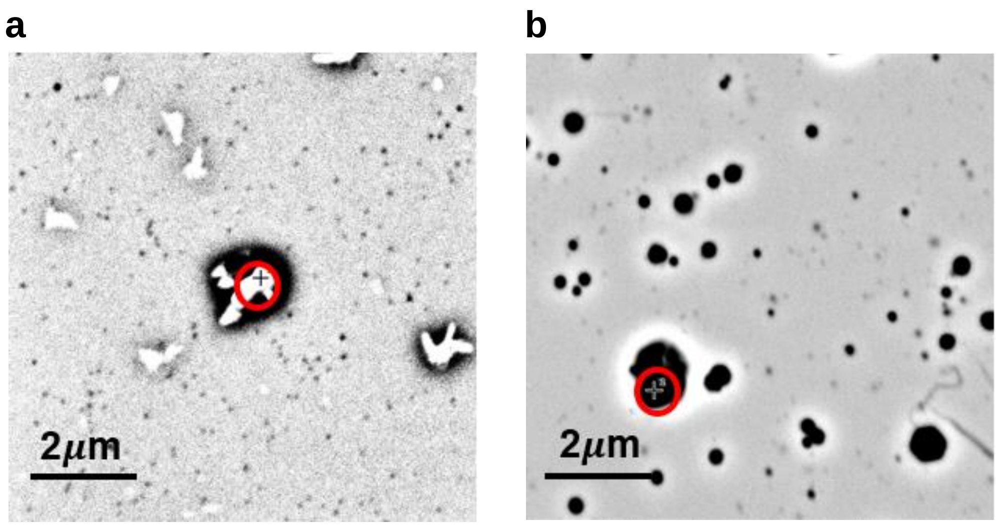

Figure S3. SEM image of (a) Sample B and (b) Sample C.

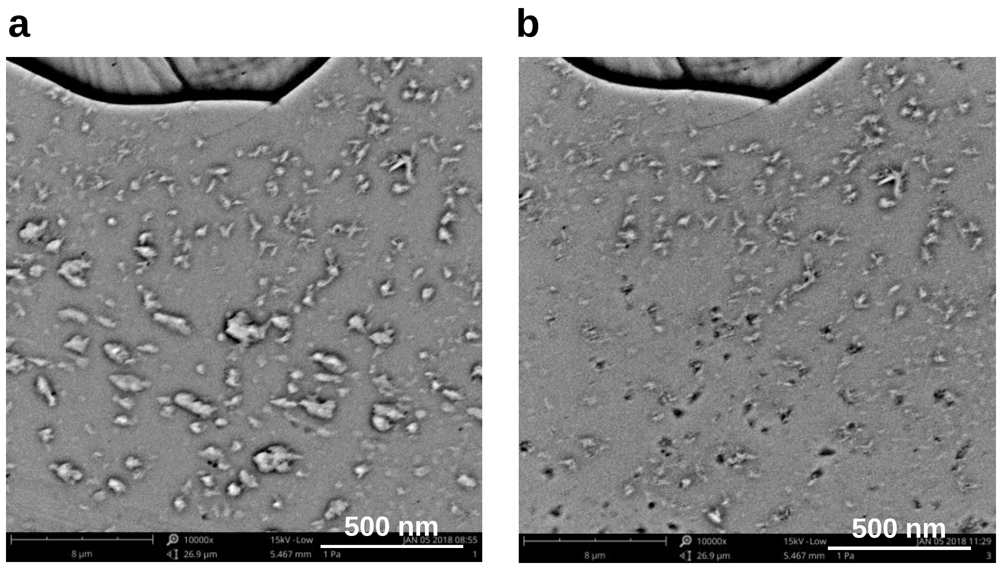

Figure S4. SEM micrographs of the surface of Sample B before (a) and after (b) HCl ($HCl:H_2O$=1:1) treatment at a temperature of 60 ℃ for 30 mins.

6. Secondary ion mass spectroscopy (SIMS) measurements for the Mg-implanted 1.5-μm-thick UID-GaN epilayer

Figure S5 shows the secondary ion mass spectroscopy (SIMS) measurements on the undoped and Mg-implanted 1.5-μm-thick UID-GaN epilayer after thermal annealing. To obtain a broad range of Mg distribution, high implantation energy and dose (250 keV at a dose of 4×10^{14} cm^{-2} and 500 keV at a dose of 6×10^{14} cm^{-2}) have been used, followed by a proper annealing process at 1230 ℃ for 30 mins in N_2, which is identical to the condition for the thick GaN epilayer (7-μm-thick) used in the fabrication of

p-i-n diode in the manuscript. It is expected that no Mg elements were detected in the undoped GaN epilayer. In comparison, the Mg concentration above 5×10^{18} cm^{-3} is distributed in the implanted sample to a depth of ~0.63 μm according to the SIMS measurement. The observed projection profile is slightly shallower than the SRIM prediction, which might be due to the outside diffusion of Mg towards the surface region during the high temperature annealing process. It indicates that besides the implanted region, a parallel conduction layer of n-type GaN with thickness of about 0.87 μm still cannot be ignored. As a result, the compensation of Mg acceptors leads to the reduction of electron concentration and high resistivity of the implanted sample even after annealing.

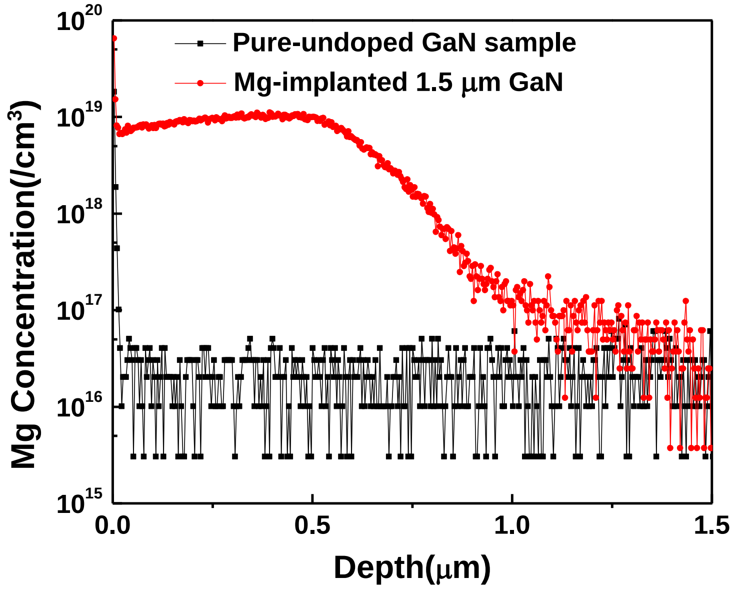

Figure S5. The secondary ion mass spectroscopy (SIMS) results for the undoped and Mg-implanted (after thermal annealing) 1.5-μm-thick GaN epilayers.

7. Hall effect and thermopower measurements

Hall effect measurements are the most popular method to evaluate carrier transport properties including the carrier type, concentration and mobility. In the Hall measurement, the probed results are true if the sample contains only one kind of carriers, i.e. either electrons or holes and the carrier transport takes place in one region of the sample. However, the measurement brings difficulty to precisely determine the transport properties in the multi-layers, in which two kinds of carriers participate the

transport and more than one conductive channels occur. Especially in this work, due to the large difference in carrier concentration, mobility and thickness of multilayers, p-type conduction, which is formed in the shallow surface region with a rather small distributed thickness and low mobility, might be compensated or covered by the stronger n-type conduction of the underneath un-implanted n-type GaN region. As a result, the directly probed carrier type, concentration and mobility by Hall measurement did not reveal the true situation. We have made great efforts in Hall measurements as suggested, and however, most of the implanted samples exhibit high resistivity, which makes the Hall measurement unstable. For instance, the Hall coefficient, which is used to determine the carrier type, is positive or negative now and then. To clarify our efforts in Hall measurement, we present comprehensive analysis as follows.

In this work, the 7-μm-thick GaN epilayer, which was used for the fabrication of p-i-n diode, consists of 1-μm unintentional doped (UID)-GaN, 2-μm Si doped n-GaN (electron concentration of ~5×10^{18} cm^{-3}), and 4-μm UID-GaN on sapphire (0001) substrate. Upon this thick GaN epilayer, the Mg ion implantation with two energy steps was performed including a beam energy of 30 keV with a dose of 1.5×10^{14} cm^{-2} and 60 keV with a dose of 2×10^{14} cm^{-2}. It gives rise to an average Mg concentration of about 5×10^{18} cm^{-3} within the projected range of 300 nm, as evidenced by the SIMS measurement result shown in Figure 3(a) in the main manuscript. As a result, due to the existence of very thick n-type conduction layer underneath the implanted layer, the confirmation of p-type implanted layer by Hall measurement is difficult, and a two-carrier transport model [S1, S2] are often employed to explain the Hall measurement data, which involves two parallel conducting channels.

As described in Ref. S1, considering a relatively thin implanted region of approximate thickness d_s on a uniform thick GaN epilayer, the theoretical Hall mobility μ_H and carrier concentration n can be written as

$$\mu_H = \frac{d_s \mu_{\mathrm{Cond},s}^2 n_s + (d - d_s)\mu_{\mathrm{Cond},b}\mu_{H,b} n_b}{d_s \mu_{\mathrm{Cond},s} n_s + (d - d_s)\mu_{\mathrm{Cond},b} n_b}$$

$$n = \frac{1}{d} \frac{[d_s \mu_{\text{Cond},s} n_s + (d - d_s) \mu_{\text{Cond},b} n_b]^2}{d_s \mu_{\text{Cond},s}^2 n_s + (d - d_s) \mu_{\text{Cond},b} \mu_{H,b} n_b} \quad \text{(Eq. S1)}$$

where d is the total thickness of the sample, μ_{Cond} is the conductivity mobility, and the subscript s or b represents the implanted or bulk part of the sample.

In our case, due to the thick n-type conduction layer (thickness: 7μm-0.3μm =6.7μm) beneath the shallow surface Mg-implanted region (thickness: 0.3μm), the Hall measurement results (μ_H and n) will be seriously distorted by the bulk part of the GaN epitaxial, which has n-type carriers with large thickness and high mobility. Additionally, the high temperature annealing process will lead to the diffusion of oxygen from sapphire substrate into GaN epilayer. As oxygen is a good donor in GaN, it will contribute electrons and increase the electron background concentration. As a matter of fact, the Hall coefficient is the average of various carriers weighted by the square of mobility and thus, even though the magnitude of holes is higher than that of electrons, the rather lower hole mobility as compared to electrons would result in the negative signal for Hall coefficient. It is similar to the case of p-type InN materials where a strong accumulation of electrons with high mobility occurred at InN surface region. In fact, people made significant efforts in Hall measurements to confirm the conversion of conduction type in Mg-implanted GaN, but few of them succeeded in the past decade. Temperature-dependent Hall-effect measurement have been attempted to explore the buried p-type conduction [S3-S4]. Thus, we have also performed the temperature-dependent Hall measurement on the implanted and undoped GaN samples. The temperature-dependent carrier concentration was plotted in Figure S6 as temperature increasing from room-temperature (RT) to 600 K. The Mg-implanted GaN and the undoped GaN sample were cut from the same as-grown wafer. It was found that the carrier concentration of the Mg-implanted GaN film after thermal annealing is lower than the undoped GaN film and the difference in electron concentration becomes larger with the elevated temperature, which is due to the compensation effect by more Mg acceptors activated at high temperature. The distinguished feature is a decrease of carrier concentration at a "kink" temperature point around 540 K. This

phenomenon differs from the classical n-type behavior in GaN, for which carrier concentration should increase with the elevated temperature due to the ionization of intrinsic shallow donors. The decrease of electron concentration is a result of the compensation by the creation of mobile holes contributed by activated Mg acceptors in the implanted region at high temperature.

Alternatively, thermopower measurement is an effective method to determine the conduction type of semiconductors as reported in [S3-S6]. It has been applied to demonstrate p-type carriers in case that Hall measurements were failed due to parallel n-type carrier systems in InN [S4]. As described in [S4], the total Seebeck coefficient can be expressed as

$$S = \frac{n_n \mu_n S_n + n_p \mu_p S_p}{n_n \mu_n + n_p \mu_p}, \qquad \text{(Eq. S2)}$$

As compared between Eq. S1 and Eq. S2, it is noted that the Seebeck coefficient is weighted by $n_i\mu_i$ instead of $n_i\mu_i^2$ in the Hall coefficient (here the subscript $i = n$ or p, corresponding to different channels). Thus, in comparison, the contribution from the channel with electrons will be decreased in the thermopower measurement. To avoid the contribution of electrons in Si-doped layer, alternatively, we have also performed the Mg-implantation on a 1.5-μm-thick UID-GaN epilayer. It was implanted with Mg ions in two energy steps: 250 keV at a dose of 4×10^{14} cm^{-2} and 500 keV at a dose of 6×10^{14} cm^{-2}. The subsequent post-annealing processes were identical to that on thick GaN epilayers. The higher implantation energy and dose were used to result in a deeper projection range. The Mg concentration above 5×10^{18} cm^{-3} is distributed to a depth of ~0.63 μm according to the SIMS measurement (Figure S5). It should be noted that the observed projection profile is slightly shallower than the SRIM prediction, which might be due to the outside diffusion of Mg towards the surface region during the high temperature annealing process. The thermopower measurement has been performed and shown in Figure 3(c) in the manuscript. For the undoped GaN, the negative Seebeck coefficient is expected due to the predominant conduction by electrons, while the positive Seebeck coefficient yields for the Mg-implanted sample, which provides a direct evidence of the existence of mobile holes and conversion

to p-type conduction. Furthermore, we have made the thermopower measurement for the 7-μm-thick GaN epitaxial that we mentioned above. However, as the sample has a 2-μm Si doped n-GaN layer with high electron concentration of ~5×10^{18} cm^{-3} and high mobility (~100 cm^2/Vs), therefore, the thermopower measurement is seriously affected by the n-type carriers in the bottom part of the sample.

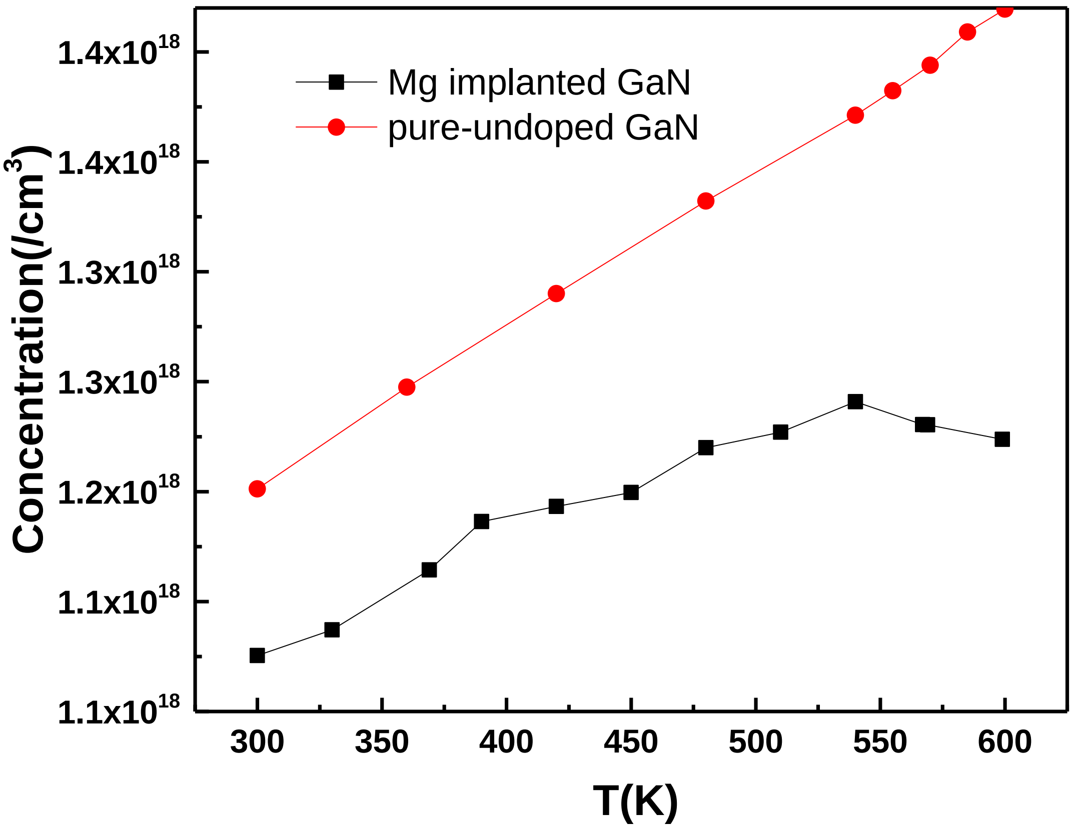

Figure S6. Temperature-dependent carrier concentration in the undoped GaN and the Mg-implanted layer after annealing.

8. P-i-n device fabrication and characterization details

To confirm the achieved p-type GaN by Mg ion implantation and optmized thermal activation, a p-i-n diode was fabricated with following processes. Firstly, following the standard lithographic processes, the topmost p-type GaN was selectively etched till the n-GaN layer with a depth of 4.5μm by inductively coupled plasma (ICP) process. During selective etching process, a 800-nm-thick metal Ni deposited by an electron beam evaporator was used as as hard mask, and then removed by HCl solution

after dry etching. Then, Ti/Al/Ni/Au (20/40/30/70 nm) metal stacks were deposited on n-GaN to form the Ohmic contact by electron beam evaporation and standard lift-off process. The metalization to form Ohmic contact was performed by rapid thermal annealing (RTA) at 850 ℃ for 30 s in nitrogen ambient. Finally, the Ohmic contact to p-GaN induced by Mg implantation and thermal activation was made by Ni/Au (30/80 nm) metal stack after a 5-min RTA metalization at 500 ℃ in atmosphere.

The current-voltage (I-V) characteristics were performed using a Keithley source meter model 2636 in dark condition. The electroluminescence (EL) characterizations were performed by using HORIBA iHR 320 and a PMT detector. The electron-beam-induced-curent (EBIC) measurements were carried out by using a FEI Helios 600 NanoLab Focused Ion Beam (dual-beam) system.

To make sure the formation of good Ohmic contact, the I-V performance has been characterized for both n- and p-type sides of the device. The results are shown in Figure S7. The I-V characteristics for both n- and p-type sides of the device are linear and uniform, thus verifying the good Ohmic contact on both sides. The I-V characteristics for UID-GaN and Mg-doped GaN have also been measured and shown in Figure S8. Both of them exhibit good Ohmic characteristics. For the sake of easy comparison, we clarify the data of Mg-doped GaN as the inset of Figure S8. It can be seen that the resistance of UID-GaN is much smaller than that of Mg-doped GaN.

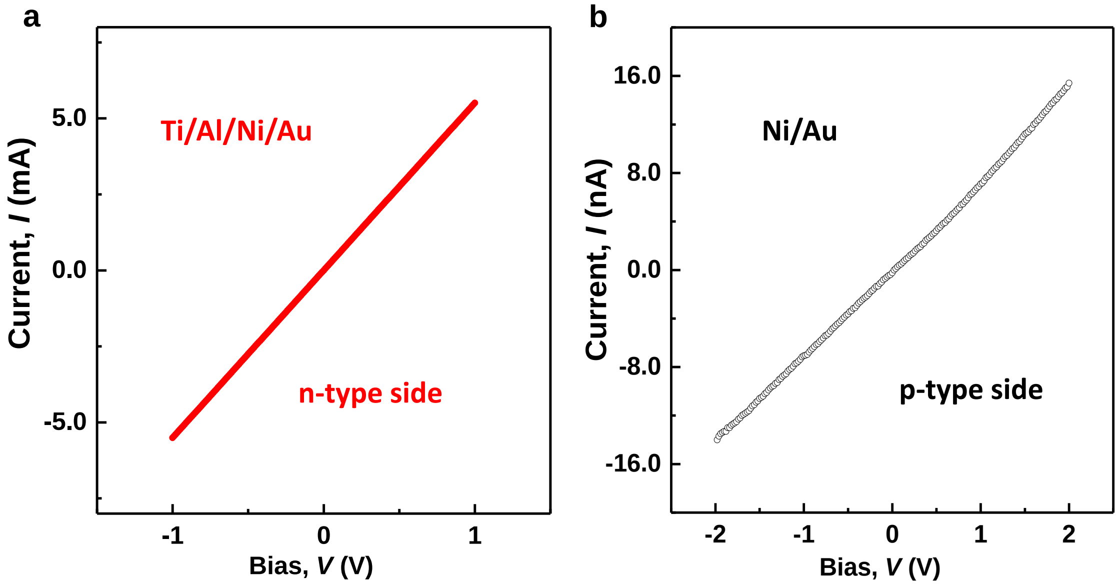

Figure S7. I-V characteristics for (a) n- and (b) p-sides of the device.

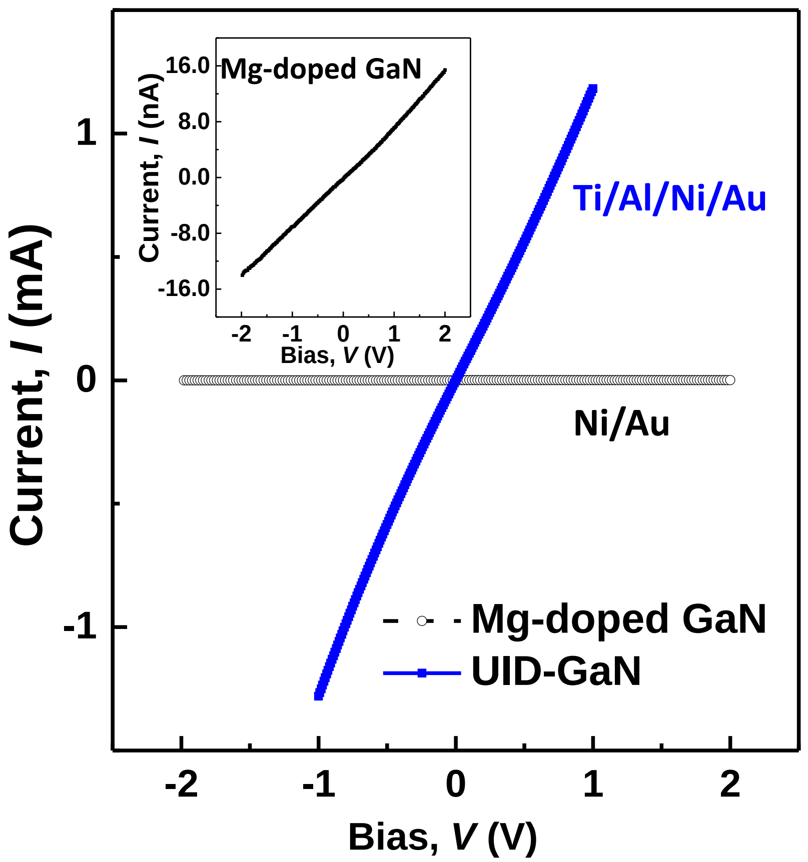

Figure S8. I-V characteristics for metallic contacts on unintentional doped GaN and Mg-doped GaN.

9. Electrical field distribution at the breakdown point

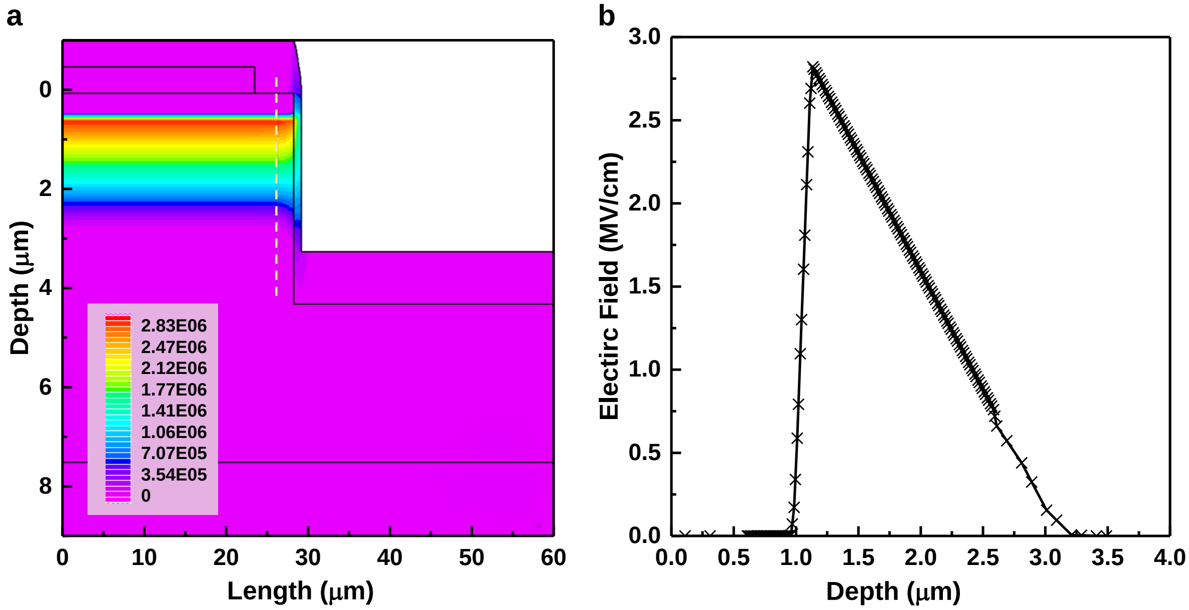

Figure S9. (a) Simulated electrical field distribution in Mg-implanted p-i-n GaN diode at a reverse bias of 300 V, and (b) the extracted E-field distribution along the white dashed cutline in panel (a).

10. Light output-current (L-I) characteristics

Figure S10 shows the results of electroluminescence (EL) intensities versus currents. The light output-current (L-I) characteristics were obtained from near-band edge UV emission around 3.26 eV. It is found that the normalized EL integrated intensity shows a superlinear dependence on the injection current and no saturation is observed even at 95 mA. The L-I characteristics can be fitted by the power law $P = cI^m$, where parameter m reflects the effect of deep-level states on the recombination process. The fitting in Figure S10 gives rise to a value of m = 2, and for the case of $m > 1$, it is resulted from the dominant non-radiative tunneling processes, i.e., carriers tunnel into the available defect states in the space-charge region followed by non-radiative recombination. It can be understood that lattice distortion cannot be fully recurred by the post-annealing processes and the remaining defects are possibly in deep-level states and affect the L-I characteristics. Nevertheless, the presence of EL UV emission together with typical LED's L-I characteristics provides a direct evidence of the formation of p-i-n LED structure.

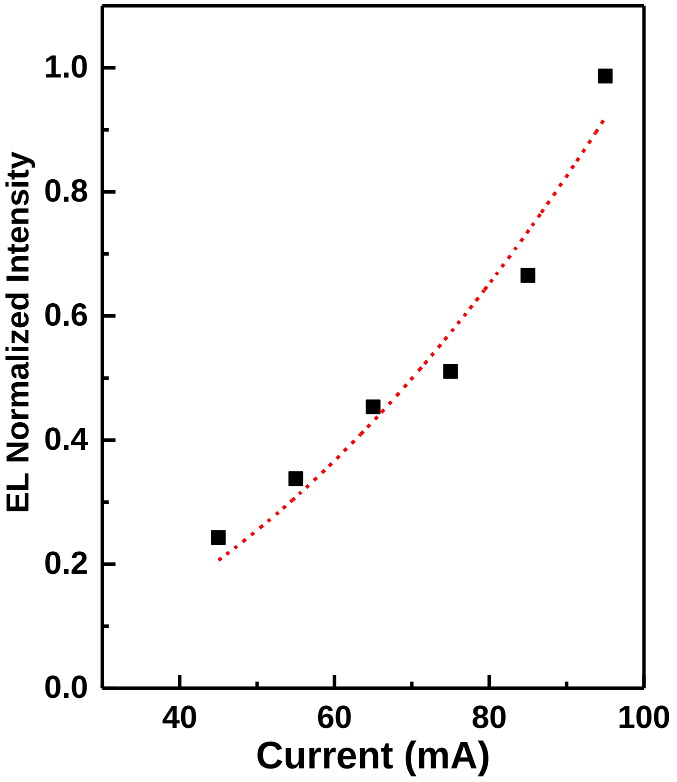

Figure S10. Light output as a function of injection current at forward bias.

11. Thermopower results of other samples

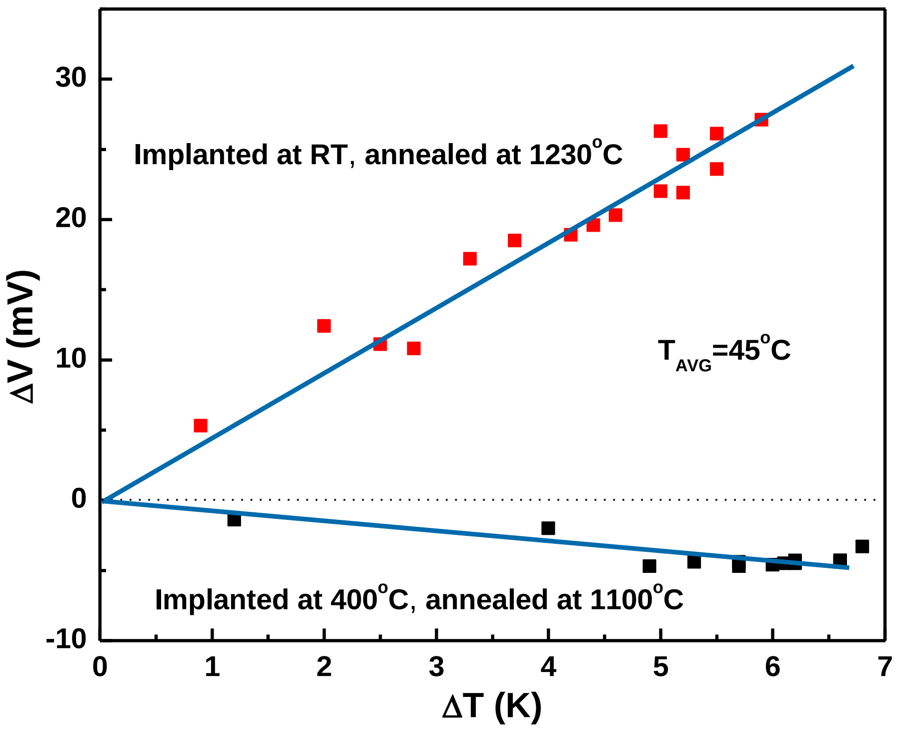

Figure S11. Thermopower results of 1.5 μm-thick GaN:Mg sample implanted at RT and annealed at 1230 ℃ (red dots), and 1.5 μm-thick GaN:Mg sample implanted at 400 ℃ and annealed at 1100 ℃ (dark dots).

References

[S1] Look, D. C. Two-layer Hall-effect model with arbitrary surface-donor profiles:application to ZnO. *J. Appl. Phys.* 104, 063718 (2008).

[S2] Kumar, Y.; Bern, F.; Barzola-Quiquia, J.; Lorite, I.; Esquinazi, P. Study of non-linear Hall effect in nitrogen-grown ZnO microstructure and the effect of H^+-implantation. *Appl. Phys. Lett.*, 107, 022403 (2015).

[S3] Papadogianni, A.; White, M. E.; Speck, J.S.; Galazka, Z.; Bierwagen, O. Hall and Seebeck measurements estimate the thickness of a (buried) carrier system: Identifying interface electrons in In-doped SnO_2 films. *Appl. Phys. Lett.* 107, 252105 (2015).

[S4] Guo, L.; Wang, X. Q.; Zheng, X. T.; Yang, X. L.; Xu, F. J.; Tang, N.; Lu, L. W.; Ge, W. K.; Shen, B.; Dmowski, L. H.; Suski, T. Revealing of the transition from n- to p-type conduction of InN:Mg by photoconductivity effect measurement. *Sci. Rep.* 4,4371 (2014).

[S5] Dmowski, L. H.; Baj, M.; Suski, T.; Przybytek J.; , Czernecki, R.; Wang, X.; Yoshikawa, A.; Lu, H.; Schaff, W. J.; Muto, D.; Nanishi Y. Search for free holes in InN:Mg-interplay between surface layer and Mg-acceptor doped interior. *J. Appl. Phys.* 105, 123713 (2009).

[S6] Ager III, J. W.; Miller, N.; Jones, R. E.; Yu, K. M.; Wu, J.; Schaff, W. J.; Walukiewicz, W. Mg-doped InN and InGaN-Photoluminescence, capacitance–voltage and thermopower measurements. *Phys. Stat. Sol. B* 245, 873–877 (2008).
